# Crystal structure of [NaZn(BTC)(H_2_O)_4_]·1.5H_2_O (BTC = benzene-1,3,5-tri­carb­oxy­l­ate): a heterometallic coordination compound

**DOI:** 10.1107/S2056989015012001

**Published:** 2015-06-27

**Authors:** Min Ni, Quanle Li, Hao Chen, Shengqing Li

**Affiliations:** a College of Science, Huazhong Agricultural University, Wuhan, Hubei 430070, People’s Republic of China

**Keywords:** crystal structure, heterometallic coordination compound, benzene-1,3,5-tri­carb­oxy­lic acid, hydrogen bonding

## Abstract

The title coordination polymer, poly[[μ-aqua-tri­aqua­(μ_3_-benzene-1,3,5-tri­carboxyl­ato)sodiumzinc] sesquihydrate], {[NaZn(C_9_H_3_O_6_)(H_2_O)_4_]·1.5H_2_O}_*n*_, was obtained in ionic liquid microemulsion at room temperture by the reaction of benzene-1,3,5-tri­carb­oxy­lic acid (H_3_BTC) with Zn(NO_3_)_2_·6H_2_O in the presence of NaOH. The asymmetric unit comprises two Na^+^ ions (each located on an inversion centre), one Zn^2+^ ion, one BTC ligand, four coordinating water mol­ecules and two solvent water molecules, one of which is disordered about an inversion centre and shows half-occupation. The Zn^2+^ cation is five-coordinated by two carboxyl­ate O atoms from two different BTC ligands and three coordinating H_2_O mol­ecules; the Zn—O bond lengths are in the range 1.975 (2)–2.058 (3) Å. The Na^+^ cations are six-coordinated but have different arrangements of the ligands: one is bound to two carboxyl­ate O atoms of two BTC ligands and four O atoms from four coordinating H_2_O mol­ecules while the other is bound by four carboxyl­ate O atoms from four BTC linkers and two O atoms of coordinating H_2_O mol­ecules. The completely deprotonated BTC ligand acts as a bridging ligand binding the Zn^2+^ atom and Na^+^ ions, forming a layered structure extending parallel to (100). An intricate network of O—H⋯O hydrogen bonds is present within and between the layers.

## Related literature   

For general background to heterometallic coordination compounds, see: Stock & Biswas (2012 ▸); Gao *et al.* (2005 ▸); Zhou *et al.* (2012 ▸). For details of the synthesis, see: Shang *et al.* (2013 ▸); Fu *et al.* (2011 ▸). For the potential application of this compound, see: Huang *et al.* (2014 ▸).
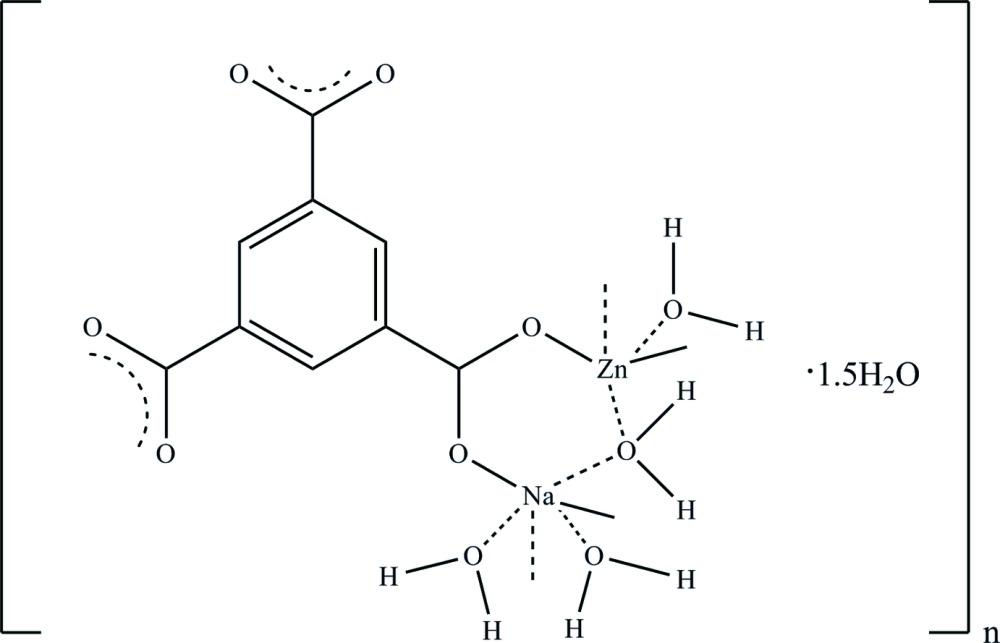


## Experimental   

### Crystal data  

[NaZn(C_9_H_3_O_6_)(H_2_O)_4_]·1.5H_2_O
*M* *_r_* = 394.56
Triclinic, 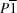

*a* = 7.0980 (11) Å
*b* = 9.8000 (16) Å
*c* = 11.2043 (17) Å
α = 66.923 (2)°
β = 73.598 (2)°
γ = 84.720 (3)°
*V* = 687.68 (19) Å^3^
*Z* = 2
Mo *K*α radiation
μ = 1.88 mm^−1^
*T* = 296 K
0.05 × 0.03 × 0.02 mm

### Data collection  

Bruker APEXII CCD diffractometer
Absorption correction: multi-scan (*SADABS*; Bruker, 2009 ▸) *T*_min_ = 0.912, *T*_max_ = 0.963
7585 measured reflections
4331 independent reflections
2567 reflections with *I* > 2σ(*I*)
*R*_int_ = 0.051

### Refinement  

*R*[*F* ^2^ > 2σ(*F* ^2^)] = 0.051
*wR*(*F* ^2^) = 0.113
*S* = 0.97
4331 reflections
214 parameters
H-atom parameters constrained
Δρ_max_ = 0.79 e Å^−3^
Δρ_min_ = −0.69 e Å^−3^

### 

Data collection: *APEX2* (Bruker, 2009 ▸); cell refinement: *SAINT-Plus* (Bruker, 2009 ▸); data reduction: *SAINT-Plus*; program(s) used to solve structure: *SHELXS7* (Sheldrick, 2008 ▸); program(s) used to refine structure: *SHELXL2013* (Sheldrick, 2015 ▸); molecular graphics: *OLEX2* (Dolomanov *et al.*, 2009 ▸); software used to prepare material for publication: *OLEX2*.

## Supplementary Material

Crystal structure: contains datablock(s) I. DOI: 10.1107/S2056989015012001/zp2017sup1.cif

Structure factors: contains datablock(s) I. DOI: 10.1107/S2056989015012001/zp2017Isup2.hkl

Supporting information file. DOI: 10.1107/S2056989015012001/zp2017Isup3.docx

**Fig. 1.**
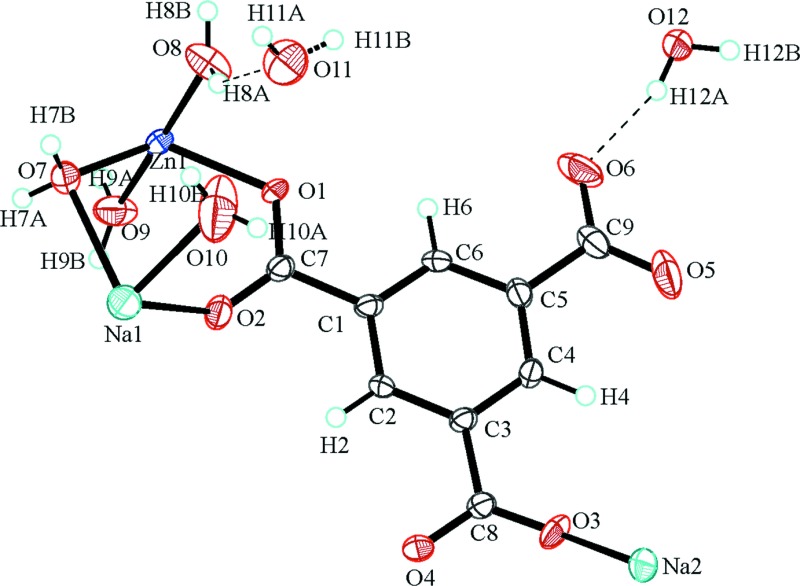
The molecular structure of the title compound with the atom-numbering scheme and 30% probability ellipsoids.

**Fig. 2.**
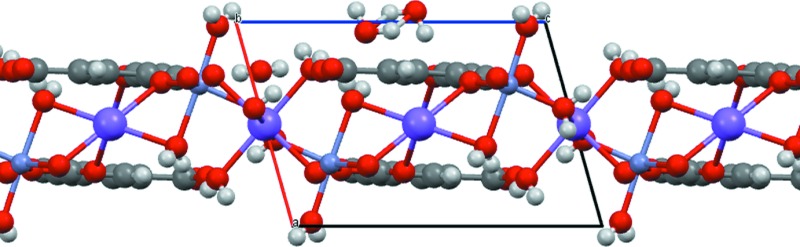
The packing diagram viewed along the b axis.

. DOI: 10.1107/S2056989015012001/zp2017fig1.tif
The mol­ecular structure of the title compound with the atom-numbering scheme and 30% probability ellipsoids.

b . DOI: 10.1107/S2056989015012001/zp2017fig2.tif
The packing diagram viewed along the *b* axis.

**Fig. 3.**
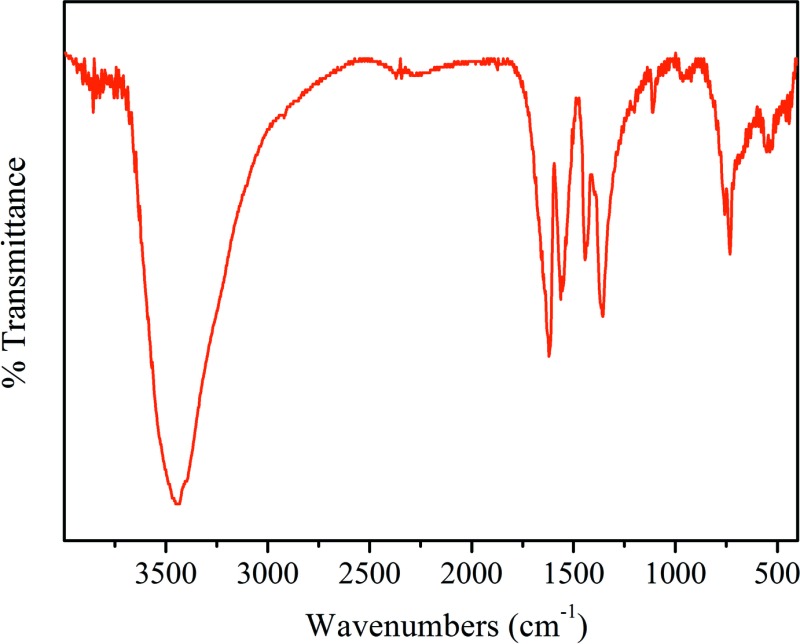
The FT–IR spectrum of the title compound.

. DOI: 10.1107/S2056989015012001/zp2017fig3.tif
The FT–IR spectrum of the title compound.

**Fig. 4.**
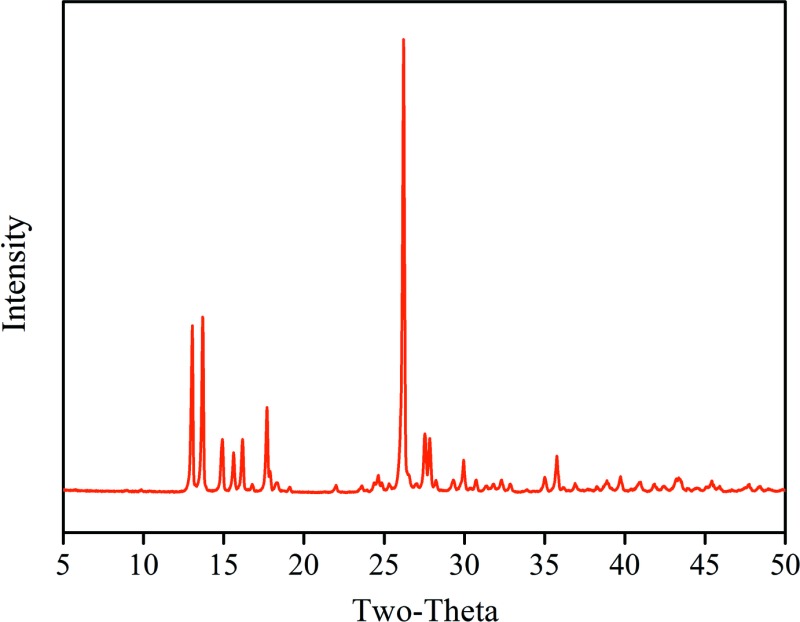
The XRD pattern of the title compound.

. DOI: 10.1107/S2056989015012001/zp2017fig4.tif
The XRD pattern of the title compound.

CCDC reference: 1055450

Additional supporting information:  crystallographic information; 3D view; checkCIF report

## Figures and Tables

**Table 1:** Hydrogen-bond geometry (, )

*D*H*A*	*D*H	H*A*	*D* *A*	*D*H*A*
O7H7*A*O5^i^	0.82	1.79	2.587(4)	162
O7H7*B*O12^ii^	0.82	1.93	2.740(4)	172
O8H8*A*O10	0.82	2.40	3.114(5)	146
O8H8*A*O11	0.82	1.98	2.672(8)	142
O8H8*B*O6^ii^	0.82	2.05	2.641(5)	128
O9H9*A*O12^iii^	0.82	1.95	2.734(4)	159
O9H9*B*O2^iv^	0.82	2.01	2.823(4)	170
O10H10*A*O5^v^	0.82	2.06	2.719(6)	137
O10H10*B*O9^vi^	0.82	2.31	3.079(5)	155
O11H11*A*O3^vii^	0.85	2.03	2.835(8)	157
O11H11*B*O3^v^	0.85	2.27	2.866(7)	127
O11H11*B*O11^viii^	0.85	1.33	1.973(9)	128
O12H12*A*O6	0.82	1.86	2.652(4)	161
O12H12*B*O4^ix^	0.82	1.97	2.787(3)	172

Symmetry codes: (i) 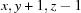; (ii) 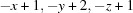; (iii) 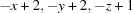; (iv) 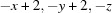; (v) 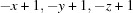; (vi) 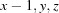; (vii) 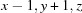; (viii) 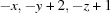; (ix) 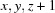.

## supplementary crystallographic information

### S1. Synthesis and crystallization

In the experiment, the microemulsion of desired composition containing water, [Bmim]PF_6_, and Triton X-100 was prepared using the method reported previously (Gao *et al.* 2005). H_3_BTC (0.210 g, 1.0 mmol), NaOH (0.040 g, 1.0 mmol) and Zn(NO_3_)_2_.6H_2_O (0.298 g, 1.0 mmol) were added one by one into the microemulsion (20 g) which was clear and transparent system including 1.444 g [Bmim]PF_6_, 10.428 g Triton X-100 and 8.310 g water. The whole system was stirred continuously for 24 h at 25^o^C. Then, the product crystals were collected by centrifugation at 4500 r/min and washed with alcohol three times (3x20 mL) to remove the surfa­ctant and [Bmim]PF_6_. Then, the crystals were dried in a vacuum oven at 60^o^C for 24 h. The resulting colorless crystals of the title compound were obtained.

### S2. Refinement

Crystal data, data collection and structure refinement details are summarized in Table 1.

### Crystal data

**Table:**

[NaZn(C_9_H_3_O_6_)(H_2_O)_4_]·1.5H_2_O	*Z* = 2
*M**_r_* = 394.56	*F*(000) = 402
Triclinic, *P*1	*D*_x_ = 1.906 Mg m^−^^3^
*a* = 7.0980 (11) Å	Mo *K*α radiation, λ = 0.71073 Å
*b* = 9.8000 (16) Å	Cell parameters from 1047 reflections
*c* = 11.2043 (17) Å	θ = 2.4–22.5°
α = 66.923 (2)°	µ = 1.88 mm^−^^1^
β = 73.598 (2)°	*T* = 296 K
γ = 84.720 (3)°	Block, colourless
*V* = 687.68 (19) Å^3^	0.05 × 0.03 × 0.02 mm

### Data collection

**Table:**

Bruker APEXII CCD diffractometer	2567 reflections with *I* > 2σ(*I*)
Radiation source: fine-focus sealed tube	*R*_int_ = 0.051
φ and ω scans	θ_max_ = 32.0°, θ_min_ = 2.3°
Absorption correction: multi-scan (*SADABS*; Bruker, 2009)	*h* = −10→10
*T*_min_ = 0.912, *T*_max_ = 0.963	*k* = −14→14
7585 measured reflections	*l* = −16→16
4331 independent reflections	

### Refinement

**Table:**

Refinement on *F*^2^	Primary atom site location: structure-invariant direct methods
Least-squares matrix: full	Secondary atom site location: difference Fourier map
*R*[*F*^2^ > 2σ(*F*^2^)] = 0.051	Hydrogen site location: mixed
*wR*(*F*^2^) = 0.113	H-atom parameters constrained
*S* = 0.97	*w* = 1/[σ^2^(*F*_o_^2^) + (0.0356*P*)^2^] where *P* = (*F*_o_^2^ + 2*F*_c_^2^)/3
4331 reflections	(Δ/σ)_max_ = 0.001
214 parameters	Δρ_max_ = 0.79 e Å^−^^3^
0 restraints	Δρ_min_ = −0.69 e Å^−^^3^

### Special details

**Table:**

Geometry. All e.s.d.'s (except the e.s.d. in the dihedral angle between two l.s. planes) are estimated using the full covariance matrix. The cell e.s.d.'s are taken into account individually in the estimation of e.s.d.'s in distances, angles and torsion angles; correlations between e.s.d.'s in cell parameters are only used when they are defined by crystal symmetry. An approximate (isotropic) treatment of cell e.s.d.'s is used for estimating e.s.d.'s involving l.s. planes.
Refinement. olex2_refinement_description 1. Fixed Uiso At 1.2 times of: All C(H) groups At 1.5 times of: All O(H,H) groups 2. Others Fixed Sof: O11(0.5) H11A(0.5) H11B(0.5) 3.a Riding coordinates: O7(H7A,H7B), O8(H8A,H8B), O9(H9A,H9B), O10(H10A,H10B), O12(H12A,H12B) 3.b Free rotating group: O11(H11A,H11B) 3.c Aromatic/amide H refined with riding coordinates: C2(H2), C4(H4), C6(H6)

### Fractional atomic coordinates and isotropic or equivalent isotropic displacement parameters (Å^2^)

**Table:**

	*x*	*y*	*z*	*U*_iso_*/*U*_eq_	Occ. (<1)
Zn1	0.69252 (6)	1.17043 (4)	0.17723 (4)	0.02302 (13)	
Na1	0.5000	1.0000	0.0000	0.0322 (5)	
Na2	0.5000	0.0000	0.5000	0.0319 (5)	
C1	0.7424 (5)	0.7068 (3)	0.3327 (3)	0.0184 (7)	
C2	0.7376 (5)	0.5950 (3)	0.2865 (3)	0.0186 (7)	
H2	0.7312	0.6192	0.1988	0.022*	
C3	0.7423 (5)	0.4476 (3)	0.3710 (3)	0.0188 (7)	
C4	0.7512 (5)	0.4130 (4)	0.5013 (3)	0.0207 (7)	
H4	0.7529	0.3140	0.5581	0.025*	
C5	0.7576 (5)	0.5231 (4)	0.5492 (3)	0.0211 (7)	
C6	0.7566 (5)	0.6703 (4)	0.4623 (3)	0.0204 (7)	
H6	0.7657	0.7455	0.4917	0.024*	
C7	0.7273 (5)	0.8667 (4)	0.2423 (3)	0.0204 (7)	
C8	0.7345 (5)	0.3229 (4)	0.3272 (3)	0.0206 (7)	
C9	0.7632 (5)	0.4837 (5)	0.6923 (4)	0.0291 (8)	
O1	0.7044 (4)	0.9607 (3)	0.2967 (3)	0.0371 (7)	
O2	0.7376 (4)	0.8999 (3)	0.1217 (3)	0.0341 (6)	
O3	0.7335 (4)	0.1931 (3)	0.4071 (3)	0.0325 (6)	
O4	0.7242 (4)	0.3558 (3)	0.2063 (2)	0.0268 (6)	
O5	0.7596 (4)	0.3477 (4)	0.7661 (3)	0.0467 (8)	
O6	0.7682 (4)	0.5863 (4)	0.7316 (3)	0.0479 (8)	
O7	0.5762 (4)	1.2363 (3)	0.0195 (2)	0.0292 (6)	
H7A	0.6483	1.2811	−0.0557	0.044*	
H7B	0.4703	1.2778	0.0249	0.044*	
O8	0.3875 (4)	1.1497 (3)	0.3061 (3)	0.0396 (7)	
H8A	0.3040	1.0995	0.3031	0.059*	
H8B	0.3305	1.2022	0.3464	0.059*	
O9	0.9800 (4)	1.1943 (3)	0.0630 (3)	0.0382 (7)	
H9A	1.0364	1.2580	0.0713	0.057*	
H9B	1.0536	1.1721	0.0026	0.057*	
O10	0.2322 (5)	0.9423 (4)	0.2021 (4)	0.0790 (13)	
H10A	0.1808	0.8593	0.2402	0.119*	
H10B	0.1445	1.0027	0.1899	0.119*	
O11	0.0497 (9)	0.9938 (8)	0.4107 (6)	0.0482 (16)	0.5
H11A	−0.0558	1.0390	0.3993	0.072*	0.5
H11B	0.0429	0.9553	0.4942	0.072*	0.5
O12	0.7580 (4)	0.6003 (3)	0.9650 (2)	0.0294 (6)	
H12A	0.7717	0.5773	0.9002	0.044*	
H12B	0.7499	0.5234	1.0318	0.044*	

### Atomic displacement parameters (Å^2^)

**Table:**

	*U*^11^	*U*^22^	*U*^33^	*U*^12^	*U*^13^	*U*^23^
Zn1	0.0340 (2)	0.01306 (19)	0.0218 (2)	−0.00099 (15)	−0.00540 (16)	−0.00759 (16)
Na1	0.0424 (13)	0.0290 (11)	0.0309 (12)	0.0027 (9)	−0.0169 (10)	−0.0128 (10)
Na2	0.0457 (13)	0.0204 (10)	0.0228 (11)	−0.0072 (9)	−0.0013 (9)	−0.0048 (9)
C1	0.0191 (16)	0.0153 (15)	0.0210 (17)	−0.0019 (12)	−0.0009 (13)	−0.0096 (13)
C2	0.0267 (18)	0.0163 (16)	0.0141 (16)	−0.0003 (13)	−0.0044 (13)	−0.0078 (13)
C3	0.0211 (17)	0.0145 (15)	0.0205 (17)	−0.0018 (12)	−0.0023 (13)	−0.0081 (13)
C4	0.0239 (18)	0.0150 (16)	0.0184 (17)	0.0016 (13)	−0.0045 (13)	−0.0025 (13)
C5	0.0217 (17)	0.0228 (17)	0.0150 (17)	0.0005 (13)	−0.0033 (13)	−0.0045 (14)
C6	0.0259 (18)	0.0190 (16)	0.0194 (17)	0.0014 (13)	−0.0039 (13)	−0.0123 (14)
C7	0.0221 (17)	0.0146 (16)	0.0221 (18)	−0.0014 (13)	−0.0017 (14)	−0.0070 (14)
C8	0.0229 (17)	0.0170 (16)	0.0221 (18)	0.0007 (13)	−0.0047 (14)	−0.0089 (14)
C9	0.0241 (19)	0.044 (2)	0.0187 (19)	0.0110 (16)	−0.0063 (15)	−0.0131 (18)
O1	0.071 (2)	0.0100 (12)	0.0212 (14)	0.0004 (12)	0.0018 (13)	−0.0062 (11)
O2	0.0607 (19)	0.0187 (13)	0.0253 (15)	0.0030 (12)	−0.0202 (13)	−0.0053 (11)
O3	0.0481 (17)	0.0124 (12)	0.0351 (16)	0.0001 (11)	−0.0128 (12)	−0.0058 (11)
O4	0.0411 (15)	0.0191 (12)	0.0235 (14)	−0.0019 (10)	−0.0079 (11)	−0.0117 (11)
O5	0.0529 (19)	0.053 (2)	0.0203 (15)	0.0040 (15)	−0.0114 (13)	0.0006 (14)
O6	0.058 (2)	0.070 (2)	0.0325 (17)	0.0229 (17)	−0.0228 (14)	−0.0346 (17)
O7	0.0337 (15)	0.0261 (14)	0.0213 (14)	0.0047 (11)	−0.0037 (11)	−0.0057 (11)
O8	0.0298 (15)	0.065 (2)	0.0303 (16)	−0.0049 (13)	−0.0034 (12)	−0.0265 (15)
O9	0.0386 (17)	0.0369 (16)	0.0418 (18)	−0.0062 (12)	0.0029 (13)	−0.0260 (14)
O10	0.059 (2)	0.062 (3)	0.068 (3)	0.0028 (18)	−0.0052 (18)	0.016 (2)
O11	0.041 (4)	0.052 (4)	0.052 (4)	0.008 (3)	−0.018 (3)	−0.019 (4)
O12	0.0440 (16)	0.0224 (13)	0.0223 (14)	−0.0001 (11)	−0.0096 (11)	−0.0086 (11)

### Geometric parameters (Å, º)

**Table:**

Zn1—Na1	3.6267 (5)	C3—C8	1.495 (4)
Zn1—Na2^i^	3.2603 (6)	C4—H4	0.9300
Zn1—O1	1.975 (2)	C4—C5	1.390 (5)
Zn1—O4^i^	2.009 (2)	C5—C6	1.390 (4)
Zn1—O7	2.013 (2)	C5—C9	1.506 (5)
Zn1—O8	2.214 (3)	C6—H6	0.9300
Zn1—O9	2.058 (3)	C7—O1	1.267 (4)
Na1—Zn1^ii^	3.6267 (5)	C7—O2	1.242 (4)
Na1—O2	2.369 (3)	C8—O3	1.235 (4)
Na1—O2^ii^	2.369 (3)	C8—O4	1.286 (4)
Na1—O7	2.529 (3)	C9—O5	1.262 (5)
Na1—O7^ii^	2.529 (3)	C9—O6	1.252 (5)
Na1—O10	2.413 (3)	O1—Na2^i^	2.482 (2)
Na1—O10^ii^	2.413 (3)	O4—Zn1^iv^	2.009 (2)
Na2—Zn1^iii^	3.2603 (6)	O7—H7A	0.8201
Na2—Zn1^iv^	3.2603 (6)	O7—H7B	0.8201
Na2—O1^iv^	2.482 (2)	O8—Na2^i^	2.403 (3)
Na2—O1^iii^	2.482 (2)	O8—H8A	0.8200
Na2—O3	2.339 (3)	O8—H8B	0.8201
Na2—O3^v^	2.339 (3)	O9—H9A	0.8199
Na2—O8^iii^	2.403 (3)	O9—H9B	0.8200
Na2—O8^iv^	2.403 (3)	O10—H10A	0.8200
C1—C2	1.389 (4)	O10—H10B	0.8200
C1—C6	1.384 (4)	O11—H11A	0.8500
C1—C7	1.509 (4)	O11—H11B	0.8500
C2—H2	0.9300	O12—H12A	0.8203
C2—C3	1.386 (4)	O12—H12B	0.8200
C3—C4	1.382 (4)		
			
Na2^i^—Zn1—Na1	108.751 (15)	O3^v^—Na2—O8^iii^	81.75 (9)
O1—Zn1—Na1	81.47 (8)	O3—Na2—O8^iii^	98.25 (9)
O1—Zn1—Na2^i^	49.45 (7)	O3—Na2—O8^iv^	81.75 (9)
O1—Zn1—O4^i^	129.50 (11)	O8^iii^—Na2—Zn1^iv^	137.24 (6)
O1—Zn1—O7	123.49 (11)	O8^iv^—Na2—Zn1^iii^	137.24 (6)
O1—Zn1—O8	83.14 (11)	O8^iii^—Na2—Zn1^iii^	42.76 (6)
O1—Zn1—O9	97.22 (11)	O8^iv^—Na2—Zn1^iv^	42.76 (6)
O4^i^—Zn1—Na1	147.26 (7)	O8^iii^—Na2—O1^iii^	69.50 (9)
O4^i^—Zn1—Na2^i^	89.66 (7)	O8^iii^—Na2—O1^iv^	110.50 (9)
O4^i^—Zn1—O7	105.44 (10)	O8^iv^—Na2—O1^iv^	69.50 (9)
O4^i^—Zn1—O8	88.15 (11)	O8^iv^—Na2—O1^iii^	110.50 (9)
O4^i^—Zn1—O9	89.49 (10)	O8^iii^—Na2—O8^iv^	180.0
O7—Zn1—Na1	42.24 (7)	C2—C1—C7	119.8 (3)
O7—Zn1—Na2^i^	131.91 (7)	C6—C1—C2	119.7 (3)
O7—Zn1—O8	86.91 (10)	C6—C1—C7	120.6 (3)
O7—Zn1—O9	95.17 (11)	C1—C2—H2	119.9
O8—Zn1—Na1	85.30 (7)	C3—C2—C1	120.1 (3)
O8—Zn1—Na2^i^	47.48 (7)	C3—C2—H2	119.9
O9—Zn1—Na1	97.50 (8)	C2—C3—C8	122.3 (3)
O9—Zn1—Na2^i^	131.02 (8)	C4—C3—C2	119.4 (3)
O9—Zn1—O8	177.20 (11)	C4—C3—C8	118.2 (3)
Zn1—Na1—Zn1^ii^	180.0	C3—C4—H4	119.3
O2—Na1—Zn1	53.49 (6)	C3—C4—C5	121.4 (3)
O2^ii^—Na1—Zn1^ii^	53.49 (6)	C5—C4—H4	119.3
O2^ii^—Na1—Zn1	126.51 (6)	C4—C5—C9	120.8 (3)
O2—Na1—Zn1^ii^	126.51 (6)	C6—C5—C4	118.3 (3)
O2^ii^—Na1—O2	180.0	C6—C5—C9	120.8 (3)
O2^ii^—Na1—O7^ii^	83.14 (8)	C1—C6—C5	121.0 (3)
O2—Na1—O7	83.14 (8)	C1—C6—H6	119.5
O2^ii^—Na1—O7	96.86 (8)	C5—C6—H6	119.5
O2—Na1—O7^ii^	96.86 (8)	O1—C7—C1	116.3 (3)
O2—Na1—O10^ii^	86.91 (11)	O2—C7—C1	120.1 (3)
O2^ii^—Na1—O10^ii^	93.09 (11)	O2—C7—O1	123.6 (3)
O2^ii^—Na1—O10	86.91 (11)	O3—C8—C3	120.1 (3)
O2—Na1—O10	93.09 (11)	O3—C8—O4	122.0 (3)
O7^ii^—Na1—Zn1	147.65 (5)	O4—C8—C3	117.9 (3)
O7—Na1—Zn1	32.36 (5)	O5—C9—C5	117.2 (4)
O7^ii^—Na1—Zn1^ii^	32.35 (5)	O6—C9—C5	118.8 (4)
O7—Na1—Zn1^ii^	147.64 (5)	O6—C9—O5	124.0 (4)
O7^ii^—Na1—O7	180.0	Zn1—O1—Na2^i^	93.35 (9)
O10^ii^—Na1—Zn1	99.48 (11)	C7—O1—Zn1	116.3 (2)
O10—Na1—Zn1^ii^	99.48 (11)	C7—O1—Na2^i^	140.1 (2)
O10—Na1—Zn1	80.52 (11)	C7—O2—Na1	130.6 (2)
O10^ii^—Na1—Zn1^ii^	80.52 (11)	C8—O3—Na2	132.2 (2)
O10—Na1—O7^ii^	89.62 (12)	C8—O4—Zn1^iv^	110.2 (2)
O10—Na1—O7	90.38 (12)	Zn1—O7—Na1	105.40 (10)
O10^ii^—Na1—O7^ii^	90.38 (12)	Zn1—O7—H7A	117.9
O10^ii^—Na1—O7	89.62 (12)	Zn1—O7—H7B	118.8
O10^ii^—Na1—O10	180.0	Na1—O7—H7A	101.6
Zn1^iii^—Na2—Zn1^iv^	180.0	Na1—O7—H7B	102.9
O1^iii^—Na2—Zn1^iv^	142.80 (5)	H7A—O7—H7B	107.7
O1^iv^—Na2—Zn1^iv^	37.20 (5)	Zn1—O8—Na2^i^	89.76 (9)
O1^iv^—Na2—Zn1^iii^	142.80 (5)	Zn1—O8—H8A	121.4
O1^iii^—Na2—Zn1^iii^	37.20 (5)	Zn1—O8—H8B	129.0
O1^iv^—Na2—O1^iii^	180.0	Na2^i^—O8—H8A	106.3
O3^v^—Na2—Zn1^iv^	123.65 (7)	Na2^i^—O8—H8B	89.2
O3—Na2—Zn1^iv^	56.35 (7)	H8A—O8—H8B	107.7
O3^v^—Na2—Zn1^iii^	56.35 (7)	Zn1—O9—H9A	109.9
O3—Na2—Zn1^iii^	123.65 (7)	Zn1—O9—H9B	141.4
O3—Na2—O1^iii^	102.11 (9)	H9A—O9—H9B	107.7
O3—Na2—O1^iv^	77.89 (9)	Na1—O10—H10A	120.4
O3^v^—Na2—O1^iii^	77.89 (9)	Na1—O10—H10B	110.7
O3^v^—Na2—O1^iv^	102.11 (9)	H10A—O10—H10B	107.7
O3—Na2—O3^v^	180.0	H11A—O11—H11B	109.5
O3^v^—Na2—O8^iv^	98.25 (9)	H12A—O12—H12B	107.7
			
C1—C2—C3—C4	0.2 (5)	C4—C5—C6—C1	2.3 (5)
C1—C2—C3—C8	179.1 (3)	C4—C5—C9—O5	−0.8 (5)
C1—C7—O1—Zn1	178.2 (2)	C4—C5—C9—O6	−179.8 (3)
C1—C7—O1—Na2^i^	−48.0 (5)	C6—C1—C2—C3	1.5 (5)
C1—C7—O2—Na1	114.6 (3)	C6—C1—C7—O1	−8.3 (5)
C2—C1—C6—C5	−2.8 (5)	C6—C1—C7—O2	171.6 (3)
C2—C1—C7—O1	170.3 (3)	C6—C5—C9—O5	178.5 (3)
C2—C1—C7—O2	−9.9 (5)	C6—C5—C9—O6	−0.4 (5)
C2—C3—C4—C5	−0.7 (5)	C7—C1—C2—C3	−177.0 (3)
C2—C3—C8—O3	−178.0 (3)	C7—C1—C6—C5	175.7 (3)
C2—C3—C8—O4	0.4 (5)	C8—C3—C4—C5	−179.6 (3)
C3—C4—C5—C6	−0.6 (5)	C9—C5—C6—C1	−177.1 (3)
C3—C4—C5—C9	178.8 (3)	O1—C7—O2—Na1	−65.6 (4)
C3—C8—O3—Na2	116.2 (3)	O2—C7—O1—Zn1	−1.6 (5)
C3—C8—O4—Zn1^iv^	−175.2 (2)	O2—C7—O1—Na2^i^	132.1 (3)
C4—C3—C8—O3	0.9 (5)	O3—C8—O4—Zn1^iv^	3.1 (4)
C4—C3—C8—O4	179.3 (3)	O4—C8—O3—Na2	−62.1 (5)

Symmetry codes: (i) *x*, *y*+1, *z*; (ii) −*x*+1, −*y*+2, −*z*; (iii) −*x*+1, −*y*+1, −*z*+1; (iv) *x*, *y*−1, *z*; (v) −*x*+1, −*y*, −*z*+1.

### Hydrogen-bond geometry (Å, º)

**Table:**

*D*—H···*A*	*D*—H	H···*A*	*D*···*A*	*D*—H···*A*
O7—H7*A*···O5^vi^	0.82	1.79	2.587 (4)	162
O7—H7*B*···O12^vii^	0.82	1.93	2.740 (4)	172
O8—H8*A*···O10	0.82	2.40	3.114 (5)	146
O8—H8*A*···O11	0.82	1.98	2.672 (8)	142
O8—H8*B*···O6^vii^	0.82	2.05	2.641 (5)	128
O9—H9*A*···O12^viii^	0.82	1.95	2.734 (4)	159
O9—H9*B*···O2^ix^	0.82	2.01	2.823 (4)	170
O10—H10*A*···O5^iii^	0.82	2.06	2.719 (6)	137
O10—H10*B*···O9^x^	0.82	2.31	3.079 (5)	155
O11—H11*A*···O3^xi^	0.85	2.03	2.835 (8)	157
O11—H11*B*···O3^iii^	0.85	2.27	2.866 (7)	127
O11—H11*B*···O11^xii^	0.85	1.33	1.973 (9)	128
O12—H12*A*···O6	0.82	1.86	2.652 (4)	161
O12—H12*B*···O4^xiii^	0.82	1.97	2.787 (3)	172

Symmetry codes: (iii) −*x*+1, −*y*+1, −*z*+1; (vi) *x*, *y*+1, *z*−1; (vii) −*x*+1, −*y*+2, −*z*+1; (viii) −*x*+2, −*y*+2, −*z*+1; (ix) −*x*+2, −*y*+2, −*z*; (x) *x*−1, *y*, *z*; (xi) *x*−1, *y*+1, *z*; (xii) −*x*, −*y*+2, −*z*+1; (xiii) *x*, *y*, *z*+1.
