# Supplementary material for: Crystal structure of [NaZn(BTC)(H2O)4]·1.5H2O (BTC = benzene-1,3,5-tri­carb­oxy­l­ate): a heterometallic coordination compound
Source: Acta Crystallogr E Crystallogr Commun. 2015 Jun 27;71(Pt 7):m143–4. doi: 10.1107/S2056989015012001 (PMC4518996; doi:10.1107/S2056989015012001)
Supplement: Supplementary file 3 [file e-71-0m143-Isup3.docx]

**S1. Experimental**

In the experiment, the microemulsion of desired composition containing water, [Bmim]PF_6_, and Triton X-100 was prepared using the method reported previously (Gao et al. 2005). H_3_BTC (0.210 g, 1.0 mmol), NaOH (0.040 g, 1.0 mmol) and Zn(NO_3_)_2_·6H_2_O (0.298 g, 1.0 mmol) were added one by one into the microemulsion (20 g) which was clear and transparent system including 1.444 g [Bmim]PF_6_, 10.428 g Triton X-100 and 8.310 g water. The whole system was stirred continuously for 24 h at 25℃. Then, the product crystals were collected by centrifugation at 4500 r/min and washed with alcohol three times (3×20 mL) to remove the surfactant and [Bmim]PF_6_. Then, the crystals were dried in a vacuum oven at 60℃ for 24 h. The resulting colorless crystals of the title compound were obtained.
