# Supplementary figures and images for: Crystal structure of [NaZn(BTC)(H2O)4]·1.5H2O (BTC = benzene-1,3,5-tri­carb­oxy­l­ate): a heterometallic coordination compound
Source: Acta Crystallogr E Crystallogr Commun. 2015 Jun 27;71(Pt 7):m143–4. doi: 10.1107/S2056989015012001 (PMC4518996; doi:10.1107/S2056989015012001)

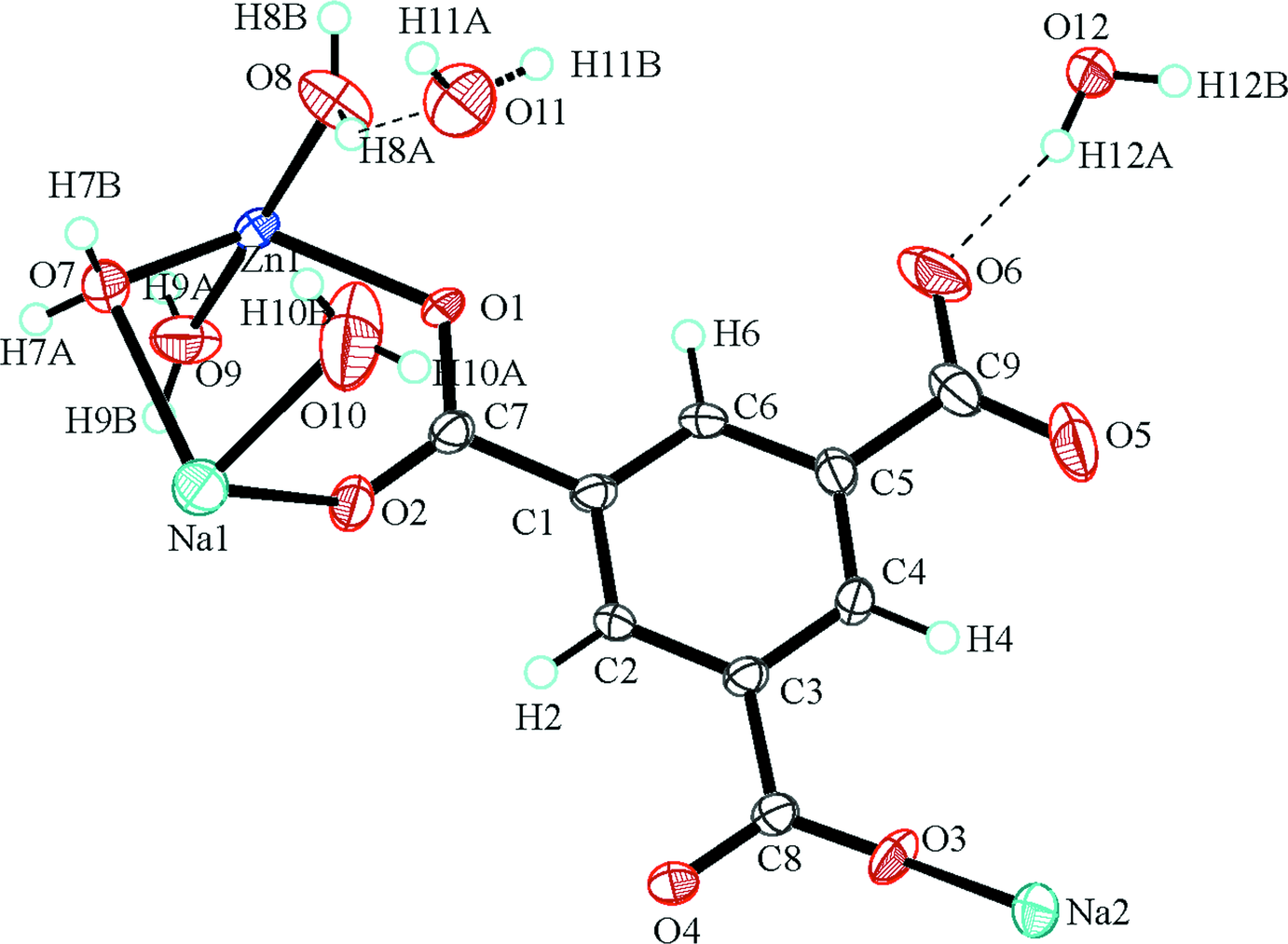

Supplement: Supplementary file 4 [file e-71-0m143-fig1.tif]

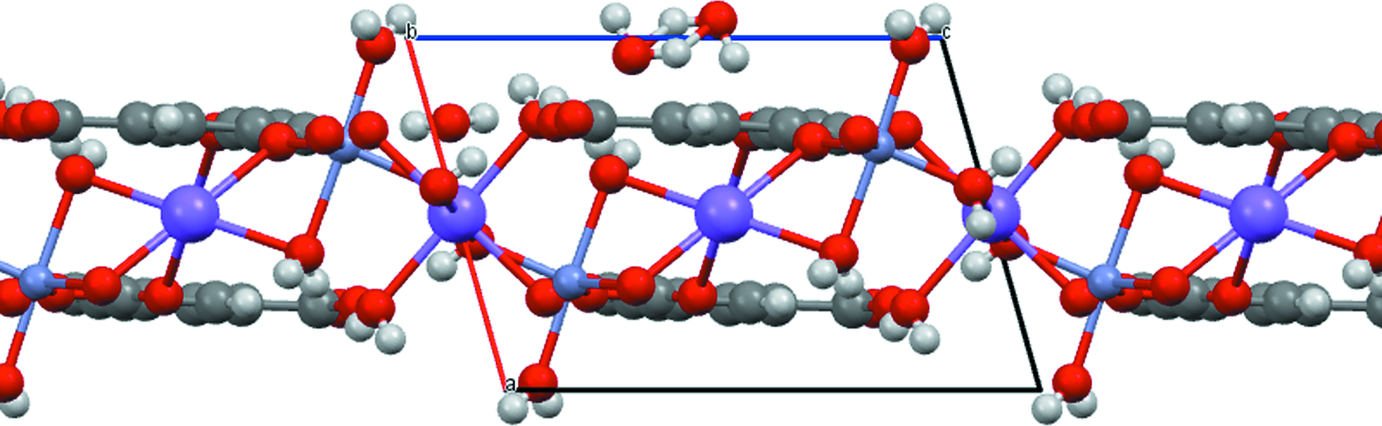

Supplement: Supplementary file 5 [file e-71-0m143-fig2.tif]

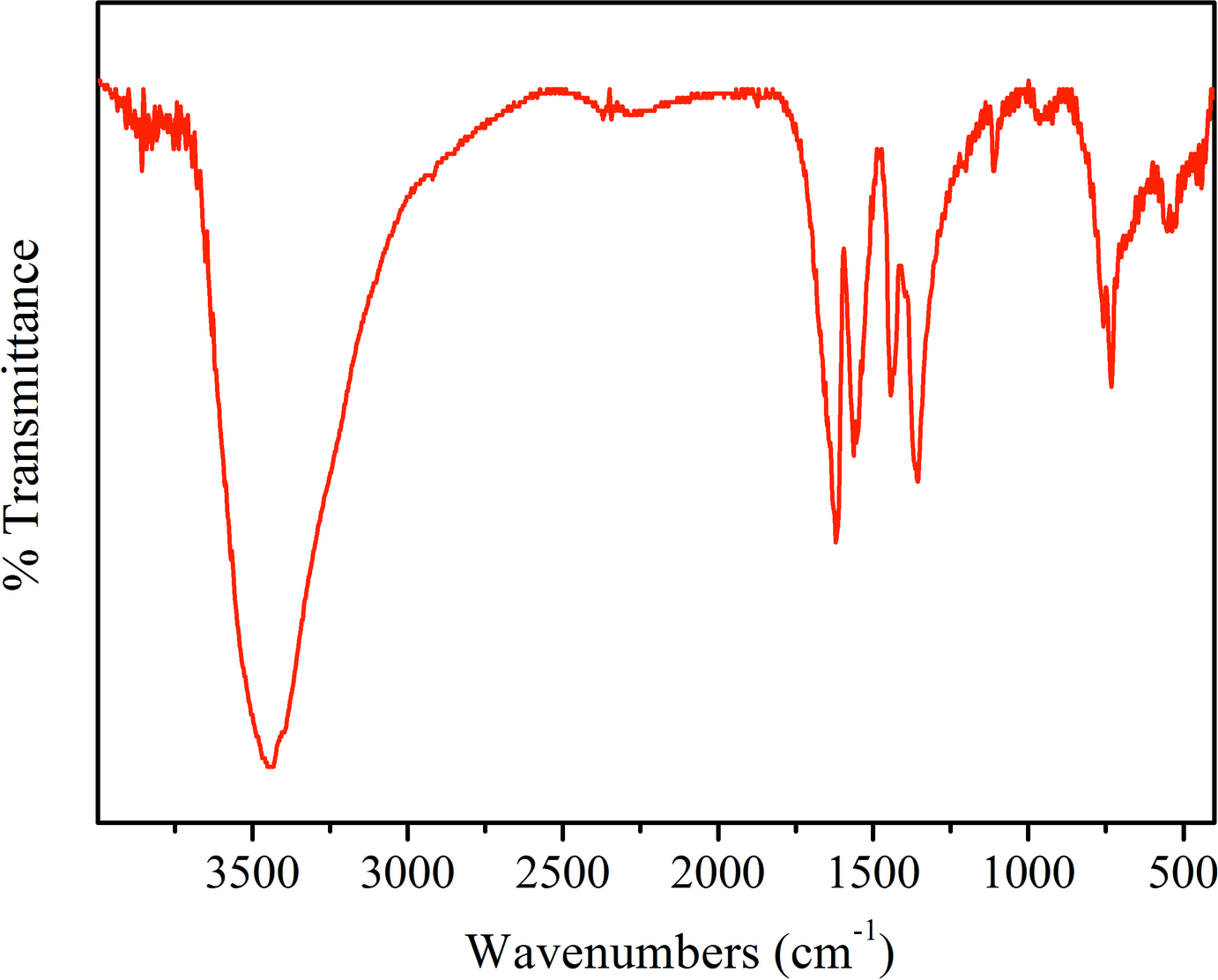

Supplement: Supplementary file 6 [file e-71-0m143-fig3.tif]

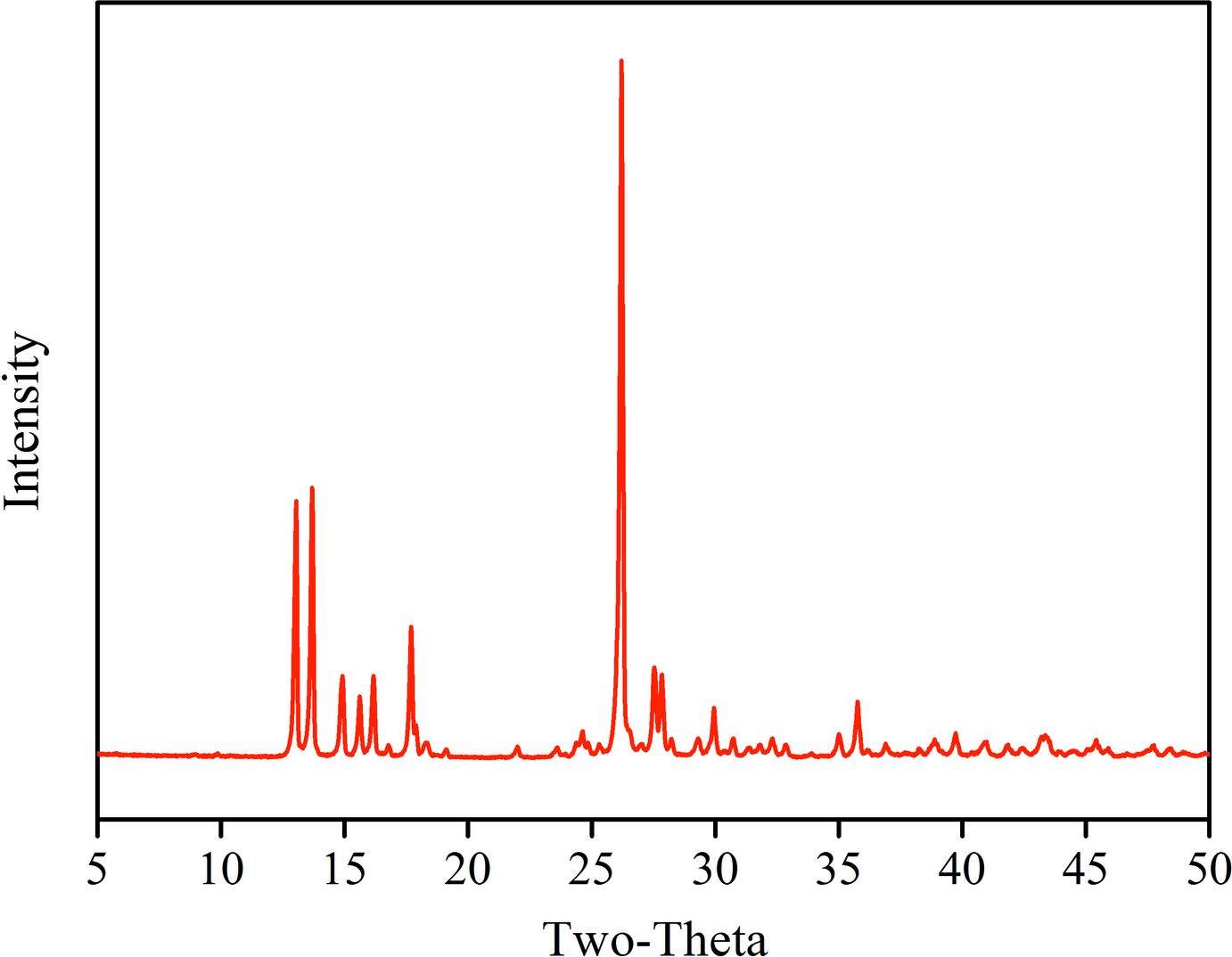

Supplement: Supplementary file 7 [file e-71-0m143-fig4.tif]
